# Beyond mismatch repair in colorectal cancer liver metastases: rethinking immunotherapy through the hepatic metastatic niche

**DOI:** 10.3389/fimmu.2026.1955959

**Published:** 2026-09-07

**Authors:** Lei Yang, Yuehua Liang, Wenqiang Wang, Jingjing Li, Bo Jia, Min Fu, Xialin Xie, Qian Chen

**Affiliations:** 1 Department of Liver Surgery, Beijing Tsinghua Changgung Hospital, School of Clinical Medicine, Tsinghua Medicine, Tsinghua University, Beijing, China; 2 Department of Radiation Oncology, Hubei Cancer Hospital, Tongji Medical College, Huazhong University of Science and Technology, Wuhan, China

**Keywords:** colorectal cancer liver metastases, hepatic metastatic niche, immune resistance, immunotherapy, mismatch repair, spatial immunology

## 1 Introduction

Immune checkpoint blockade has made deficient mismatch repair/microsatellite instability-high (dMMR/MSI-H) status the principal biomarker for immunotherapy in metastatic colorectal cancer. With more than 5 years of follow-up, KEYNOTE-177 continues to show durable benefit from first-line pembrolizumab, and CheckMate 8HW has established nivolumab plus ipilimumab as a highly active option in the same molecular subgroup (1–3). In contrast, checkpoint inhibitors are not standard therapy for unselected proficient mismatch repair/microsatellite-stable (pMMR/MSS) disease (4). MMR/MSI is therefore a clinically established proxy for tumor-intrinsic immunogenic potential, but effective immunity additionally depends on antigen presentation, persistence of tumor-reactive lymphocytes, and their access to malignant cells.

**Figure 1:**
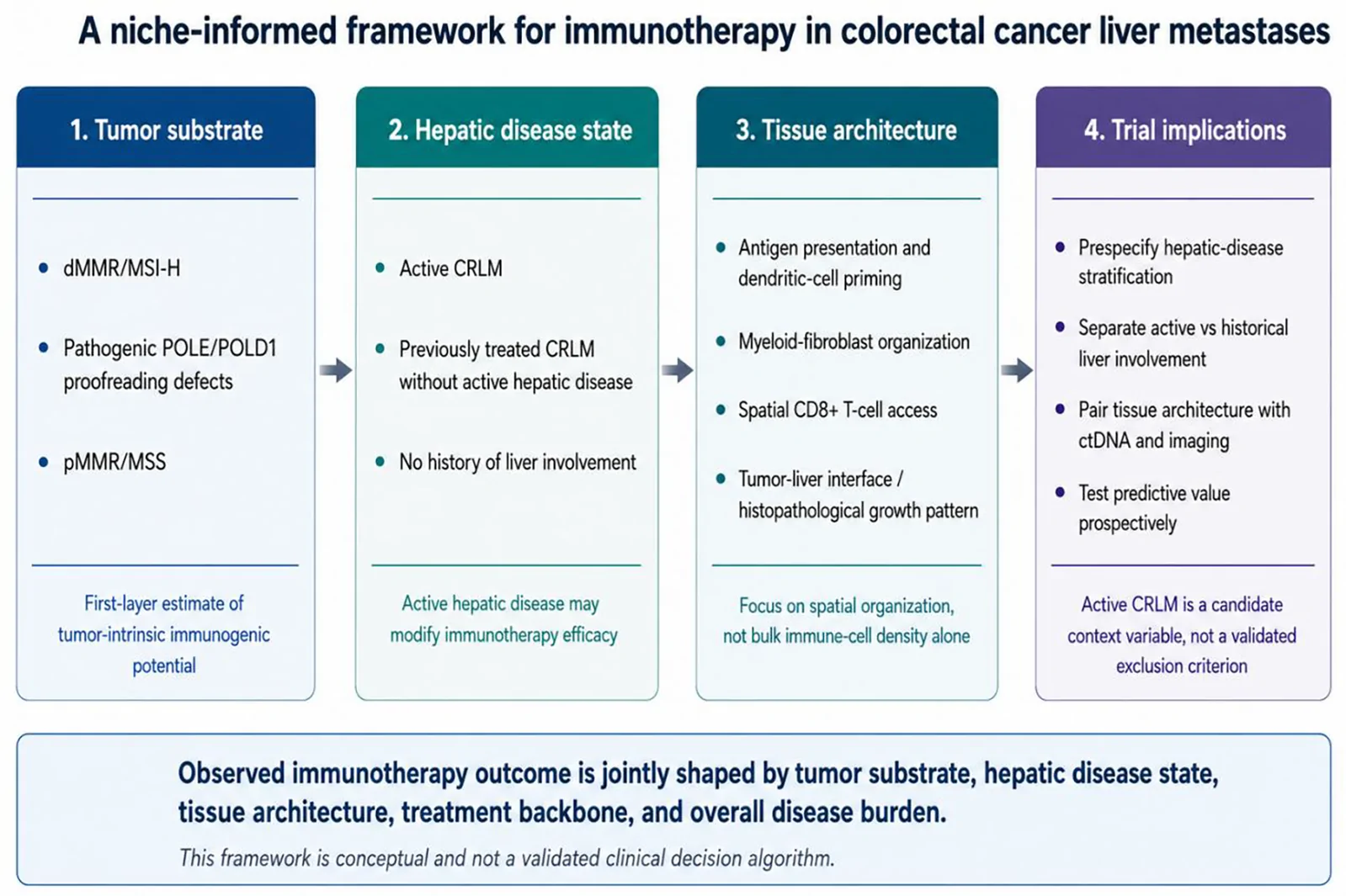
A niche-informed framework for immunotherapy in colorectal cancer liver metastases. Immunotherapy outcome in colorectal cancer liver metastases (CRLM) may reflect the combined effects of tumor-intrinsic immunogenic potential, hepatic disease state, tissue architecture, treatment backbone, and overall disease burden. MMR/MSI status and bona fide POLE/POLD1 proofreading deficiency provide an initial assessment of tumor-intrinsic immunogenic potential. Hepatic involvement should be classified as active CRLM, previously treated CRLM without active hepatic disease, or no history of liver involvement. Mechanistic assessment should extend beyond bulk immune-cell density to include dendritic-cell priming, myeloid–fibroblast organization, spatial CD8+ T-cell access, and the tumor–liver interface. Prospective studies should prespecify hepatic-disease stratification and integrate tissue, imaging, and circulating tumor DNA analyses. This framework is conceptual and does not constitute a validated clinical decision algorithm. CRLM, colorectal cancer liver metastases; MMR, mismatch repair; MSI, microsatellite instability.

Clinical series have associated liver involvement with inferior outcomes during checkpoint-based treatment in pMMR/MSS colorectal cancer and in dMMR/MSI-H disease, although findings are inconsistent (5, 6). We therefore use a two-domain model: MMR/MSI status and bona fide POLE/POLD1 proofreading deficiency estimate tumor-intrinsic immunogenic potential, whereas active hepatic disease may constrain priming, effector-cell survival, and tumor-cell access (**Figure 1**). The framework is intended to organize testable hypotheses rather than serve as a clinical decision rule. Its central test is whether hepatic disease status adds predictive information beyond genotype, treatment backbone, total disease burden, and patient selection.

We regard active hepatic disease as an adverse clinical context and a potential modifier of immunotherapy efficacy rather than a contraindication to treatment. We focus on mechanisms and study designs that can separate a treatment-specific liver interaction from prognosis, tumor burden, and treatment selection.

## 2 What MMR explains—and what it does not

The benefit of checkpoint blockade in dMMR/MSI-H disease supports the view that mutation-derived neoantigens and pre-existing immune recognition provide a therapeutically exploitable substrate (1–3). Nonetheless, dMMR/MSI-H tumors are not uniformly responsive. A pooled retrospective analysis of 104 patients receiving first-line checkpoint inhibition associated liver metastasis with lower response and shorter progression-free survival (5). A smaller series reported high response rates despite liver involvement and instead highlighted lesion number, peritoneal disease, and tumor mutational burden (6). The discordance argues against withholding immunotherapy solely because of hepatic disease.

In pMMR/MSS disease, several datasets show a similar association, but most are single-arm or retrospective. A cohort study and a *post hoc* analysis of CCTG CO.26 linked liver metastases to lower disease control or less checkpoint-related benefit (7, 8). Responses in regorafenib-nivolumab and regorafenib-ipilimumab-nivolumab studies were concentrated in patients without active liver disease (9–11). Botensilimab-balstilimab likewise produced no responses among 24 efficacy-evaluable patients with active liver metastases, whereas responses occurred after prior liver resection or ablation (12). However, patients whose hepatic disease had already been completely treated constitute a selected subgroup, so the observed difference cannot be attributed to removal of the hepatic niche alone.

Tumor burden is an essential alternative explanation. A liver-specific effect is more credible if hepatic status remains informative after accounting for burden and hepatic lesions fare worse than comparable nonhepatic lesions. Burden should include lesion count, summed diameter or volume, extrahepatic burden, and the hepatic fraction of disease; within-patient lesion-level response offers an additional control, although lesion size and prior local therapy still matter. Existing evidence only partly meets this standard. In a nonhypermutated cohort receiving PD-1-based combinations, liver metastasis and baseline lesion number were independently associated with durable clinical benefit, and hepatic lesions were less responsive than lung, nodal, or peritoneal lesions (13). Pakvisal et al. analyzed 132 MSS/pMMR mCRC patients treated with ICI-based regimens, including 93 with active liver metastases at ICI initiation (14). Clinical benefit was 16.1% with versus 46.2% without active liver disease; median PFS was 2.1 versus 2.5 months (HR 1.68, 95% CI 1.13-2.51) and median OS was 6.17 versus 11.53 months (HR 2.03, 95% CI 1.35-3.06). In multivariable models using active liver metastases as reference, no history of liver metastases remained associated with longer PFS (adjusted HR 0.04, 95% CI 0.006-0.23) and OS (adjusted HR 0.006, 95% CI 0.00-0.07), whereas prior complete metastasectomy did not (PFS adjusted HR 1.51; OS adjusted HR 0.73). The study used multivariable Cox models but did not report matched or propensity-score sensitivity analyses; small subgroups and retrospective treatment selection leave substantial residual confounding. Randomized evidence is mixed: AtezoTRIBE showed no treatment-by-liver-metastasis interaction, whereas LEAP-017 was negative overall (15–17). Active CRLM thus remains a candidate treatment-effect modifier, not an established predictive biomarker. Rare pMMR tumors with bona fide POLE/POLD1 proofreading deficiency can remain checkpoint-sensitive (18).

## 3 Why active hepatic disease may alter immunotherapy efficacy

The liver maintains specialized immune homeostasis despite continuous exposure to portal antigens through coordinated activity of sinusoidal endothelial cells, Kupffer cells, recruited macrophages, dendritic cells, stellate cells, and lymphocytes (19). Colorectal metastases can co-opt these regulatory programs and therefore do not develop in an immunologically neutral organ.

Preclinical studies identify several nonexclusive mechanisms. Liver metastases can induce macrophage-mediated elimination of activated tumor-specific CD8-positive T cells, with effects beyond the hepatic lesion (20). In colorectal cancer liver-metastasis models, an interleukin-10-dependent regulatory T-cell-myeloid circuit increased myeloid PD-L1 expression and impaired CD8-positive T-cell immunity (21). Orthotopic pMMR CRLM models showed scarce functional dendritic cells and defective T-cell activation; restoring dendritic-cell abundance improved checkpoint-blockade activity (22). The hepatic niche may therefore impair both response generation and effector-cell survival.

Human studies provide complementary spatial evidence. Single-cell profiling identified metabolically active MRC1-positive CCL18-positive macrophages and treatment-associated immune shifts (23). Matched primary and metastatic samples showed enrichment of SPP1-positive macrophages with exhausted T-cell, regulatory T-cell, and dendritic-cell states (24), while spatial analysis linked SPP1-positive macrophage-fibroblast networks to restricted lymphocyte access in MSS CRLM (25). Direct comparison of MSS liver, lung, and peritoneal metastases found more intratumoral lymphocytes, antigen-presenting cells, APC-T-cell interactions, and lymphoid aggregates in lung lesions, whereas liver lesions showed stronger suppressive signatures (26). These data support organ-specific immune architecture without implying that every liver lesion is immune-deserted. Organ comparisons require matching for lesion size, treatment exposure, and sampling method; paired metastases can reduce interpatient molecular confounding.

These data explain why bulk immune-cell density can mislead. CD8-positive cells confined to invasive margins or stroma are not equivalent to lymphocytes contacting malignant glands, and macrophage abundance requires phenotypic and spatial context. Informative assessment captures topology, cell-cell organization, and interlesional heterogeneity. Imaging-pathology registration, multisite sampling when feasible, and documentation of the biopsied lesion can reduce single-specimen misclassification.

## 4 From organ-level association to lesion-level biology

Histopathological growth patterns offer a practical bridge between routine pathology and spatial immunology. Desmoplastic and replacement-type metastases differ in lymphoid, myeloid, fibroblast, angiogenic, and metabolic features (27, 28). Automated whole-slide classification can identify these patterns with high discriminatory performance (29). Their established value is prognostic; predictive value for immunotherapy remains unproven. Nonetheless, growth patterns demonstrate that the tumor-liver interface is biologically nonuniform and provide a testable explanation for divergent outcomes among patients with similar hepatic burden.

This lesion-level perspective argues against empiric escalation of combination therapy. Local interventions are not biologically interchangeable, and apparent benefit may reflect debulking or patient selection rather than immune reprogramming. In the 2025 EORTC ILOC phase II study, partial radiofrequency ablation or SBRT combined with durvalumab and tremelimumab produced no objective responses in untreated metastases (30). Because the study was single-arm and was not designed to compare RFA with SBRT, it cannot establish equivalence between the two modalities. Mechanistic claims require paired tissue, modality-specific sampling, and an interpretable comparator.

## 5 Implications for trial design and perioperative studies

Future studies need to separate active CRLM, previously treated CRLM without active hepatic disease, and no history of liver involvement. We define active CRLM as radiologically evident viable hepatic disease at ICI initiation, including residual or recurrent disease after liver-directed treatment. The previously treated category is descriptive, not causal: complete local treatment selects for resectability, lower burden, treatment responsiveness, and survival to immunotherapy. Retrospective analyses should anchor hepatic status to the common time zero of ICI initiation and adjust or match for total burden, metastatic sites, performance status, prior response, and systemic therapy. Reports should specify the interval from liver-directed treatment to ICI, intervening therapy, and the reason for local treatment. Sensitivity analyses restricted to comparable disease states are useful, but randomized studies or prespecified prospective cohorts remain preferable.

Biomarker programs benefit from integrating tumor substrate, tissue architecture, and longitudinal disease status (**Table 1**). MMR/MSI and bona fide POLE/POLD1 proofreading deficiency, supported by a compatible mutational signature and ultramutated phenotype, estimate tumor-intrinsic immunogenic potential. Paired tissue can assess dendritic-cell programs, macrophage-fibroblast states, CD8-positive T-cell localization, and histopathological growth pattern. ctDNA is useful for residual-disease risk but does not identify the mechanism of immune resistance; biomarker analyses need to distinguish prognosis, prediction, pharmacodynamics, and mediation.

**Table 1:** Proposed niche-informed framework for immunotherapy studies in colorectal cancer liver metastases.

Domain	Candidate variables and readouts	Interpretation	Implication for trial design
Tumor-intrinsic immune potential	MMR/MSI status; bona fide POLE/POLD1 proofreading deficiency with a compatible mutational signature and ultramutated phenotype; tumor mutational burden	Estimates tumor-intrinsic immunogenic potential; does not show that tumor-reactive immunity can be initiated or maintained within hepatic lesions	Retain molecular selection as the first domain; distinguish proofreading-deficient tumors from variants of uncertain significance or non-signature variants (1–4, 18)
Hepatic disease state	Active CRLM at treatment initiation; completely treated CRLM without viable hepatic disease; no history of liver involvement; lesion number and volume; liver-limited versus multiorgan disease	Separates current viable hepatic disease from historical involvement and helps disentangle site biology from selection and total disease burden	Prespecify hepatic disease status for stratification; do not interpret the previously treated group as a causal comparator without burden and selection adjustment; test treatment-by-liver-status interactions in randomized studies (5–17)
Antigen presentation and priming	Dendritic-cell abundance and activation; antigen-presenting-cell programs; T-cell clonality; paired baseline and on-treatment tissue	Checkpoint blockade has limited substrate when productive priming is absent	Require pharmacodynamic evidence of improved antigen-presenting-cell programs rather than inferring priming from radiographic response (22)
Myeloid and regulatory networks	SPP1-positive macrophages; MRC1-positive CCL18-positive macrophages; regulatory T cells; interleukin-10/myeloid PD-L1 signaling	Myeloid and regulatory circuits may eliminate or suppress activated effector cells	Stratify mechanistic analyses by tissue phenotype and verify cellular-state change on treatment (20, 21, 23–25)
Spatial immune access	CD8-positive cells in tumor nests versus invasive margin or stroma; macrophage-fibroblast proximity; extracellular-matrix organization	Immune-cell presence does not establish tumor-cell contact or cytotoxic function	Use multiplex imaging or spatial profiling; report the sampled lesion and prior local therapy (25, 26)
Tumor-liver interface	Desmoplastic versus replacement histopathological growth pattern; vessel co-option; adjacent liver response	Captures distinct immune, stromal, angiogenic, and metabolic architectures	Standardize growth-pattern scoring in resection studies; treat predictive value as unproven (27–29)
Dynamic disease status	Pathological regression; ctDNA clearance or persistence; imaging; paired blood and tissue	Distinguishes molecular disease control from tissue mechanism	Use ctDNA for residual-disease risk and surveillance; do not interpret ctDNA change alone as evidence of immune-niche modification (31–34)
Treatment context	Checkpoint monotherapy; immunochemotherapy; anti-VEGF or multikinase combinations; resection; thermal ablation; radiotherapy; arterial embolization/TACE	The same hepatic phenotype may have different implications across systemic backbones and local-treatment modalities	Analyze local modalities separately; do not extrapolate site effects across regimens or presume local-systemic synergy; retain standard oncological indications (15–17, 30, 35–37)

CRLM, colorectal cancer liver metastases; ctDNA, circulating tumor DNA; MMR, mismatch repair; MSI, microsatellite instability; PD-L1, programmed death-ligand 1.

Perioperative dMMR studies provide an important benchmark for tumor-intrinsic checkpoint sensitivity, although they do not test the hepatic metastatic niche. In NICHE-2, 109 of 111 patients with locally advanced dMMR colon cancer had a pathological response to short-course nivolumab plus ipilimumab; 95% achieved major pathological response and 68% pathological complete response, with no recurrence at a median 26 months (31). NICHE-3 reported 97% pathological response, 92% major pathological response, and 68% pathological complete response among 59 patients receiving nivolumab plus relatlimab (32). NEOPRISM-CRC reported a 59% pathological complete response rate among evaluable high/medium-TMB tumors treated with neoadjuvant pembrolizumab (33). Together, these studies provide a high-response benchmark in localized dMMR CRC but do not resolve whether active CRLM modifies ICI efficacy.

Resectable CRLM is informative because tissue can be obtained before treatment and at surgery. The randomized phase II PURPLE window-of-opportunity trial assigns patients to two preoperative cycles of atezolizumab plus tiragolumab or immediate surgery and incorporates pathological regression, immune infiltration, metabolic imaging, and ctDNA dynamics (34). Prespecified pharmacodynamic endpoints are important: mechanism-consistent activity could include increased CD8-positive T-cell penetration, restored antigen-presenting-cell programs, or disruption of suppressive macrophage-fibroblast organization.

After CRLM resection, persistent perioperative or postoperative ctDNA consistently predicts early recurrence and inferior recurrence-free survival (35, 36). Longitudinal tumor-informed monitoring can identify recurrence before imaging and may facilitate repeat local treatment in selected patients (37). ctDNA is therefore a marker of residual disease and recurrence risk, not evidence by itself that the hepatic niche has been remodeled. Current guidelines continue to base immunotherapy on established molecular indications and liver-directed treatment on oncological and technical criteria (4, 38).

## 6 Discussion

MMR/MSI should remain the foundation of immunotherapy selection in metastatic colorectal cancer. The unresolved question is whether active hepatic disease adds treatment-predictive information after genotype, treatment backbone, total tumor burden, and patient selection are accounted for. Many checkpoint-treated cohorts show an adverse association with liver involvement, but the available evidence does not yet establish a liver-specific resistance phenotype.

Local treatment requires modality-specific interpretation. Resection removes viable tumor and reduces burden, but selection and perioperative inflammation complicate causal inference. Thermal ablation produces coagulative necrosis; in a small prospective CRLM study, RFA and resection generated different early cytokine and chemokine profiles (39). SBRT creates a distinct radiation-injury pattern, and the ILOC experience supports evaluating it separately (30). TACE combines regional chemotherapy with arterial embolization; randomized evidence versus systemic chemotherapy in CRLM remains of very low certainty (40). Immune effects are therefore best studied with modality-specific paired sampling rather than extrapolated across interventions. Current clinical data do not establish reliable local-systemic ICI synergy (30, 38–40).

Prospective testing is needed to determine whether active hepatic disease modifies immunotherapy effect after rigorous burden adjustment. Separate analyses should test whether spatial features improve prediction beyond MMR/MSI and conventional staging and whether individual local modalities produce reproducible immune changes beyond debulking and selection. Current spatial atlases largely use post-chemotherapy resection specimens, whereas ICI cohorts often contain heavily pretreated unresectable disease; prospective baseline and on-treatment sampling is needed to connect tissue maps with response. Adequately powered randomized analyses showing no treatment-by-active-CRLM interaction, or spatial measures adding no predictive information beyond genotype and burden, would argue against the hypothesis.
